# Effect of head motion-induced artefacts on the reliability of deep learning-based whole-brain segmentation

**DOI:** 10.1038/s41598-022-05583-3

**Published:** 2022-01-31

**Authors:** Péter Kemenczky, Pál Vakli, Eszter Somogyi, István Homolya, Petra Hermann, Viktor Gál, Zoltán Vidnyánszky

**Affiliations:** 1 Brain Imaging Centre, Research Centre for Natural Sciences, Budapest, 1117 Hungary; 2 Institute of Nuclear Techniques, Budapest University of Technology and Economics, Budapest, Hungary

**Keywords:** Mathematics and computing, Computer science

## Abstract

Due to their robustness and speed, recently developed deep learning-based methods have the potential to provide a faster and hence more scalable alternative to more conventional neuroimaging analysis pipelines in terms of whole-brain segmentation based on magnetic resonance (MR) images. These methods were also shown to have higher test–retest reliability, raising the possibility that they could also exhibit superior head motion tolerance. We investigated this by comparing the effect of head motion-induced artifacts in structural MR images on the consistency of segmentation performed by FreeSurfer and recently developed deep learning-based methods to a similar extent. We used state-of-the art neural network models (FastSurferCNN and Kwyk) and developed a new whole-brain segmentation pipeline (ReSeg) to examine whether reliability depends on choice of deep learning method. Structural MRI scans were collected from 110 participants under rest and active head motion and were evaluated for image quality by radiologists. Compared to FreeSurfer, deep learning-based methods provided more consistent segmentations across different levels of image quality, suggesting that they also have the advantage of providing more reliable whole-brain segmentations of MR images corrupted by motion-induced artifacts, and provide evidence for their practical applicability in the study of brain structural alterations in health and disease.

## Introduction

Quantitative analysis of brain structure requires the accurate segmentation of cortical and subcortical brain areas and is essential to neuroimaging research and the study of various brain disorders^1–3^. In general, manual delineation of the different brain structures is prohibitively expensive and time-consuming. To alleviate this problem, several neuroimaging processing pipelines have been developed that allow for the automatic segmentation of brain magnetic resonance imaging (MRI) scans, such as FreeSurfer^4^, FSL^5^, or SPM^6,7^. For example, FreeSurfer implements the automatic parcellation of the cortical surface as well as the segmentation of subcortical structures in T1-weighted MR images using a probabilistic atlas^8,9^. FreeSurfer is a widely used segmentation tool in basic and clinical research^10^. However, a main drawback of FreeSurfer and similar automated segmentation tools is that the processing of each individual brain scan lasts for hours. This is practically infeasible to scale for large datasets given the hardware equipment of most research sites, or to apply it in a clinical setting where rapid decision making is required.

More recently, deep learning-based methods have been developed for the segmentation of medical images. Convolutional neural networks (CNNs) can be trained end-to-end to represent the image at increasing levels of abstraction, without the need for hand-engineered features^11^. CNNs are now widely adopted in the computer vision community due to their capability to achieve outstanding performance in image classification tasks^12,13^. Fully convolutional networks (FCNs), which consist entirely of convolutional layers, are capable of processing an arbitrarily-sized image and output an image of corresponding size, which makes them suitable for performing image segmentation tasks^14^. U-Net, a modified FCN architecture consisting of symmetric contracting and expanding paths, enables the precise segmentation of biomedical images even with few training data^15^.

Deep learning methods have the potential to speed up whole-brain segmentation as well. A deep neural network that has been trained offline can segment brain scans in minutes instead of hours. Nevertheless, this approach has its difficulties as well. In particular, the complex 3D structure of the input image poses a serious challenge to deep learning-based segmentation tools. In fact, applying a state-of-the-art FCN directly to a T1-weighted MRI scan with a resolution of 1 × 1 × 1 mm^3^ is highly impractical due to the memory limitations of currently available graphical processing units (GPUs). Downsampling the images prior to segmentation may result in the loss of information about the fine-grained boundaries of cortical structures. For these reasons, several different approaches have been adopted to ease the computational burden of processing high-resolution 3D images for the purpose of whole-brain segmentation. One way to tackle this problem is to make networks process 3D patches one at a time instead of the whole 3D volume at once. Recently, a Bayesian fully convolutional network, trained on non-overlapping subvolumes of T1 images, has been used effectively to predict 50-class FreeSurfer segmentations in minutes^16^. Another approach is to work on 2D slices. For example, QuickNAT^17^ consists of three FCNs, each having an architecture similar to that of U-Net and processing axial, coronal, and sagittal slices. The predictions of the three networks are combined in a view-aggregation step, based on a weighted average of predicted class probabilities, to provide the final whole-brain segmentation result. QuickNAT inspired the architecture of FastSurferCNN^18^, which uses a sequence of neighboring slices as input and is capable of segmenting the whole brain into 95 classes (with FreeSurfer segmentation as the ground truth) in 1 min on a single GPU. FastSurferCNN is integrated into FastSurfer, an image processing pipeline that performs cortical surface reconstruction and thickness analysis based on the output of FastSurferCNN, thus providing a full FreeSurfer alternative^18^.

On the whole, the studies reviewed above suggest that deep learning-based segmentation methods constitute a faster and more scalable alternative to traditional neuroimaging processing pipelines in terms of whole-brain segmentation. Besides, there is evidence regarding differences in the reliability of the two approaches. FastSurferCNN has been shown to exhibit higher test–retest reliability in the estimation of the volumes of subcortical structures than FreeSurfer^18^. Similarly, using brain scans from the Test–Retest Dataset^19^, QuickNAT has been shown to be more consistent in lateral ventricular and subcortical structural volume estimation for repeated measurements of the same subjects than FreeSurfer^17^. However, the authors also observed that FreeSurfer was more reliable in the estimation of cerebral white matter volume compared to QuickNAT, and when they were compared using a more challenging dataset in which repeated scans were acquired using different hardware at different sites^20^, the two methods showed comparable performance^17^. Taken together, these results show that deep-learning based brain segmentation can achieve comparable and often higher test–retest reliability than FreeSurfer.

In light of the above, not only speed, but reliability should also be taken into account when considering the relative advantages of these two approaches to brain segmentation, at least under certain circumstances. A hitherto uninvestigated aspect of deep learning-based brain segmentation methods is the extent to which motion-induced artifacts affect their reliability in comparison to more traditional neuroimaging processing pipelines. Patient motion affects MR image quality and often results in artifacts such as blurring and ghosting^21,22^. Due to the wide variety of imaging techniques and motion types that can occur during scanning, there is no universal methodological solution to the problem of motion-induced artifacts in MRI, but a range of mitigation and correction methods are available with variable degrees of efficacy^23^. This is obviously a limiting factor when investigating brain structural alterations in health and disease, especially when studying movement disorders and various neurological and neuropsychiatric conditions associated with an increased tendency to move, such as Parkinson’s disease^24^ or autism spectrum disorder^25^. Head motion demonstrably affects cortical gray matter volume and thickness estimates derived using the commonly used neuroimaging analyses software packages, namely FreeSurfer, SPM, and FSL^26^. In the study of Reuter et al., motion-related artifacts did not simply increase the variance of volume and thickness measures, but systematically reduced the estimated values, even after removing the most artifact-corrupted images by employing a manual quality control procedure. The authors concluded that instead of indicating a failure of the aforementioned processing pipelines, these results suggest that head motion results in image artifacts mimicking cortical atrophy that cause a bias in volume and thickness estimates^26^. To our knowledge, no systematic investigation has been performed regarding whether the reduction of MR image quality due to head motion affects the reliability of traditional and deep learning-based brain segmentation methods similarly.

In the present study, we examined whether motion-induced artifacts in MR images affect the consistency of whole-brain segmentation performed by FreeSurfer and several deep learning-based methods to a similar extent. To this end, we collected T1-weighted structural MRI brain scans from a large sample of participants (N = 110) under rest and under two active head motion conditions in which subjects were required to nod their heads either 5 or 10 times upon the presentation of a visual cue. The resulting images were divided into three categories corresponding to different degrees of image quality (clinically good/medium/bad), based on the ratings of five radiologists. We investigated several different deep learning models, namely FastSurferCNN^18^, the Bayesian neural network proposed by^16^, referred to as Kwyk, and our newly developed deep convolutional neural network for whole-brain segmentation, called ReSeg. These three models have different architectures and represent different approaches to segment 3D volumes, thus allowing for the examination of whether the reliability of deep learning methods compared to FreeSurfer depends on choice of method, that is, hyperparameters related to neural network architecture, optimization and regularization strategies. First, we assessed the performance of these models using the segmentation masks generated by FreeSurfer for the good quality images as ground truth. This analysis amounts to the estimation of the generalizability of the deep learning models, since none of the newly acquired images were used in their training. Second, using several metrics for evaluating image segmentations, we quantified the consistency between the segmentations generated for the different quality images and used statistical tests to compare these measures between FreeSurfer and each of the deep learning-based methods. Finally, we also compared the test–retest reliability between FreeSurfer and the deep learning models using a different set of brain scans included in the Test–Retest Dataset^19^.

## Material and methods

In this section, we first specify the ReSeg brain segmentation pipeline in detail and describe FastSurferCNN, Kwyk, and FreeSurfer briefly. By Kwyk, we refer to the network trained using spike-and-slab dropout and n = 3 sampling, introduced in the paper *Knowing What You Know in Brain Segmentation Using Bayesian Deep Neural Networks* by^16^. Then, we describe the datasets, evaluation metrics, and the overall strategy used for testing the generalizability and comparing the reliability of the different whole-brain segmentation methods.

### Segmentation methods

#### ReSeg

**Figure 1:**
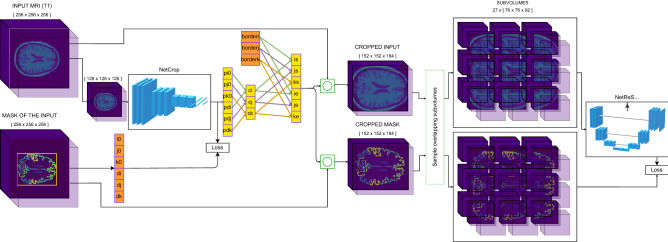
Schematic illustration of the ReSeg image processing pipeline consisting of two convolutional neural networks responsible for defining a bounding box around the input MRI volume (Net_Crop_) and performing subsequent whole-brain segmentation on the cropped volume (Net_ReSeg_). Net_Crop_ is trained to predict the coordinates of a specific vertex point (pi_0_, pj_0_, pk_0_) and the lengths of the edges along the i, j, and k axes (pd_i_, pd_j_, and pd_k_, respectively) of the bounding box circumscribing the brain in the input MRI volume. The target output vector [i_0_, j_0_, k_0_, d_i_, d_j_, d_k_] for each image is computed from the FreeSurfer mask. The coordinates of the final bounding box were determined using the center point (c_i_, c_j_, c_k_) of the bounding box predicted by Net_Crop_ and fixed lengths (border_i_, border_j_, border_k_) that had been defined based on the morphometric characteristics of adult human brains. The starting and end points of this bounding box along the i, j, and k axes are denoted by i_s_, j_s_, k_s_ and i_e_, j_e_, k_e_, respectively. This bounding box was applied to the input MRI volume and, during training, to the corresponding FreeSurfer mask (denoted by green circles). Cropped input volumes and masks were used to train the segmentation network Net_ReSeg._ During inference, only MRI volumes are cropped and segmented. The figure was created with diagrams.net. JGraph Ltd: diagrams.net (Version 15.2.9) [Software].

The pipeline consists of two consecutive steps. In the first step, a neural network (Net_Crop_) defines a bounding box around the brain in the input MRI volume, and the extracranial regions of the volume are cropped based on the coordinates of this bounding box. This step reduces the computational requirements of the subsequently applied segmentation network by removing a large, unlabeled part of each input volume. It also ensures that each volume that is fed to the segmentation network has the same shape. In the second step, the segmentation network (Net_ReSeg_; see Table 1 in Appendix) outputs a segmentation mask by labelling every voxel according to the brain region it belongs to—i.e., it performs semantic segmentation (Fig. 1).

The input of the pipeline X ∈ ℝ ^256×256×256^ is the pre-processed T1 weighted MRI volume with 1 × 1 × 1 mm^3^ resolution and the output $\widehat{Y}$ is a label array which has the shape as X. The target output Y is the segmentation mask produced by FreeSurfer based on the Desikan-Killiany (DK) atlas^27^, containing 50 brain regions similarly to the segmentation target used by Kwyk (for details, see Table 2 in Appendix).

##### Net_Crop_

**Figure 2:**
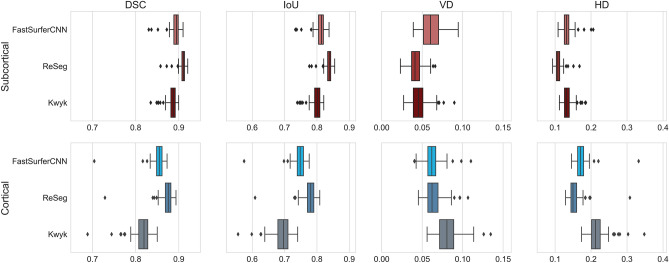
Boxplots showing the distributions of the dice similarity coefficient (DSC), intersection over union (IoU), volumetric difference (DF), and Hausdorff distance (HD) for the deep learning-based segmentation methods. Evaluation metrics were calculated to compare segmentation masks generated by the deep learning-based segmentation methods to those generated by FreeSurfer.

The core of the first step in the ReSeg pipeline is the neural network Net_Crop_ that is trained to predict the parameters of the bounding box that circumscribes the brain. The input image array X is resampled by a factor of 0.5 using spline interpolation to obtain X’ ∈ ℝ^128×128×128^, which was fed to Net_Crop_. Resizing the input array helps to reduce the computational requirements of the network. The target output vector [i_0_, j_0_, k_0_, d_i_, d_j_, d_k_] for each image is computed from the FreeSurfer mask, with i_0_, j_0_, and k_0_ denoting the coordinates of a specific vertex point of the bounding box along the i, j, and k axes, respectively, and d_i_, d_j_, and d_k_ denoting the lengths of the edges of the bounding box along the i, j, and k axes, respectively. The edges of the bounding box are parallel to the edges of the input volume. Net_Crop_ was trained to predict an approximation of this target output vector. From the output vector of Net_Crop_, the center point (c_i_, c_j_, c_k_) of the bounding box was calculated. Then, a new bounding box was defined using (c_i_, c_j_, c_k_) as the center point. The size of this bounding box was 18.4 cm in the anterior–posterior direction and 15.2 cm in superior-inferior and lateral directions. These sizes were determined based on the morphometric characteristics of adult brains^28^ and were used to guarantee that, after cropping the input image array X, a sufficiently large subvolume is preserved that contains the brain tissue in its entirety (for examples, see Figs. 1 and 2 in Appendix). Furthermore, using the same-sized bounding box for each input image ensured that each volume that is fed to the segmentation network has the same shape. Thus, after cropping the input image array X and label array Y by the bounding box, we got the arrays X_C_ and Y_C_, respectively, both of shape 152 × 152 × 184.

With regard to the architecture of Net_Crop_, it is a deep convolutional neural network containing only convolutional and dense layers but no pooling layer. It consists of 16 convolutional layers followed by 2 hidden dense layers and an output layer. The hidden layers apply Swish activation (1) on the cell outputs.

1 $$Swish\left(x\right)=x*sigmoid(x)$$

There is evidence showing that Swish tends to work better on deeper neural networks than ReLU/Leaky ReLU^29^. Dimension reduction along the height-width-depth axes is performed by the convolutional layers instead of pooling layers. Each convolutional layer uses L1L2 regularization (l1 = 0.01, l2 = 0.01) on its parameter set. The network was trained using Adam optimizer with exponentially dropping learning rate (with formula (2), where lr0 = 10e − 4; r = 0.92; s = 10).

2 $${exp\_decay}_{lr0;r;s}(epoch)=lr0* {r}^\frac{epoch}{s}$$

The target function of the optimizer is the mean squared error (MSE) between the network output and the bounding box parameter vector.

##### Net_ReSeg_

In the second step of the ReSeg pipeline, the neural network Net_ReSeg_ performs the segmentation of the cropped input image X_C_. Because 3D segmentation is computationally expensive, both X_C_ and the label array Y_C_ are split into smaller subvolumes, and these subvolumes are fed to the network one-by-one. The SAME padding that is used in convolutional networks and applied in Net_ReSeg_ causes boundary uncertainty on the edges of the network outputs, therefore, we sampled overlapping subvolumes from the arrays. The shape of each subvolume is $\frac{152}{2}$ × $\frac{152}{2}$ × $\frac{184}{2}$ and the sampling step size is [$\frac{152}{4}$, $\frac{152}{4}$, $\frac{184}{4}$], resulting in a total of 3*3*3 = 27 input subvolumes for each image.

As online data augmentation, X_C_ and Y_C_ were rotated with a probability of 0.3. The offset of the rotation (in voxels) changed randomly in the interval [− 2, 2] ∈ ℤ, and the degrees of rotation were also sampled randomly from the [− 1, 1] ∈ ℝ interval.

When the pipeline predicts the segmented brain mask, the network performs the same steps as in the case of the training pipeline until the last step. Net_ReSeg_ predicts the brain region probabilities for each of the voxels in all the subvolumes, then the logits are merged by adding and normalizing the overlapping parts of the 27 subvolumes. After this, the edges of $\widehat{c}$ are padded with the "Unknown" label (Table 2 in Appendix), using the knowledge about the size and center point coordinates of the bounding box, to get $\widehat{Y}$.

Regarding the architecture of Net_ReSeg_, it is a convolutional network inspired by U-Net^15^. It consists of an encoder and a decoder part and concatenates the layers in the two modules using skip connections. It contains only convolutional and batch normalization layers, and, similarly to Net_Crop_, performs dimension reduction using the convolutional layers instead of pooling layers. The network was trained with the RMSprop algorithm optimizing the weighted sum of Focal loss^30^, with parameters α = 4 and γ = 2, and Generalised Dice Loss (GDL;^31^). Focal loss is a modified version of cross-entropy error developed for extreme class imbalance. Because the volumetric size of the different brain regions may largely differ, it is an optimal loss function for the problem. Generalized Dice overlap also tries to eliminate the class imbalance, however, while focal loss uses hyperparameters to tackle the issue, GDL uses the number *n* of voxels classified as label *l* to weigh the loss function.

#### FastSurferCNN

FastSurferCNN is a convolutional neural network architecture that is capable of segmenting a 3D brain volume into 95 classes in under 1 min on a single GPU^18^. It consists of 3 fully convolutional networks that operate on orthogonal 2D slices, followed by the aggregation of the different views. Each FCN consists of an encoder and a decoder part including competitive dense blocks^32,33^ that induce competition between feature maps in a memory-efficient way. When segmenting a 2D slice, each FCN is provided information about the larger anatomical context by feeding a series of neighboring slices to the network as well. FastSurferCNN is integrated into the FastSurfer pipeline that performs cortical surface reconstruction based on the output of FastSurferCNN, thus providing an alternative to FreeSurfer. The segmentation target for FastSurferCNN was the brain mask produced by FreeSurfer according to the Desikan–Killiany–Tourville (DKT) atlas^34^. To evaluate the generalizability and reliability of FastSurferCNN in the present study, labels denoting the same brain structure in the left and right hemispheres were merged and a single label was assigned to all ventricles so that the final set of segmentation labels was similar to the one used by Kwyk and ReSeg (See Table 3 in Appendix for the mapping between the original FreeSurfer-DKT labels and the new labels used in the present study). Note that there remain differences between the segmentation targets of the different deep learning methods—however, the aim of the present study was not to compare these methods directly, but to examine their reliability at variable levels of image quality.

#### Kwyk

The architecture of the Bayesian deep neural network introduced by^16^ is similar to that of MeshNet^35,36^, consisting of layers including volumetric dilated convolutions^37^ that allow for the efficient processing of 3D inputs using relatively few parameters. The model was trained on non-overlapping subvolumes of 3D brain images using a novel spike-and-slab dropout that learns the dropout probability for each filter and an individual uncertainty for each weight as well. The segmentation target for Kwyk was the 50-regions brain mask produced by FreeSurfer according to the Desikan-Killiany atlas (see Table 2 in Appendix).

#### FreeSurfer

FreeSurfer is a suite of tools widely used in the processing of neuroimaging data to analyse the functional and structural properties of the human brain^10^. FreeSurfer implements automatic cortical surface reconstruction and subcortical structure segmentation using a probabilistic atlas^8,9^. In this study, all datasets were processed using FreeSurfer 6.0. For each record, we used FreeSurfer to automatically generate two brain segmentation masks; one corresponding to the Desikan-Killiany atlas, and another corresponding to the Desikan-Killiany-Tourville atlas. Some of the labels were merged as described previously in order to match the segmentation targets of deep learning methods. For the final sets of segmentation labels used for evaluating the reliability of segmentation masks produced by FreeSurfer according to the DK and DKT atlases, see Tables 2 and 3 in Appendix, respectively.

### Datasets

#### Data used for ReSeg training and evaluation

The data that was used for the training, validation, and evaluation of the ReSeg pipeline were collected from several publicly available datasets containing T1-weighted structural MRI records, namely UK Biobank^38^, ADNI^39^, SLIM^40^, and OASIS3^41^. The age and gender characteristics of the participants in this bulk dataset are displayed in Table 1. The bulk dataset was split into training, validation, and evaluation sets with the proportion of records being 0.75 (1472 records), 0.15 (315 records), and 0.15 (316 records), respectively (Table 2, for more details about these sets, see Tables 4–6 in Appendix). Note that some of the subjects have multiple records from different sessions and thus may have records in different subsets. The validation dataset was used to optimize certain hyperparameters of the networks Net_Crop_ and Net_ReSeg_, such as the learning rate and the size of the subvolumes, and the evaluation set was used to select the best layer structures for the networks. Good quality images from the Head Motion dataset were used as an independent test set to estimate the generalizability of the ReSeg pipeline (see “Generalizability”).

**Table 1:** Characteristics of the datasets used for the training, validation, and evaluation of the ReSeg brain segmentation pipeline.

Dataset	Number of records	Number of subjects	Mean age ± standard deviation (years)	Number of male subjects/records	Number of female subjects/records
UK Biobank	780	780	60.07 ± 6.72	375/375	405/405
OASIS3	61	56	72.57 ± 7.49	22/24	34/37
SLIM	620	453	20.69 ± 1.40	198/282	255/338
ADNI	642	473	74.38 ± 8.20	221/295	252/347
Total	2013	1762	53.38 ± 22.66	816/976	946/1127

**Table 2:** Number of records belonging to the sets used for the training, validation, and evaluation of the ReSeg brain segmentation pipeline.

Dataset	Number of records in the training set	Number of records in the validation set	Number of records in the evaluation set
UK Biobank	560	107	113
OASIS3	41	11	9
SLIM	411	112	97
ADNI	460	85	97
Total	1472	315	316

#### Head motion dataset

We collected a dataset in our own lab, specifically tailored to meet the requirements of analyzing the effects of ringing artifacts caused by head motion in structural MRI processing pipelines. This dataset was used to assess the generalizability and reliability of the different segmentation methods when different levels of motion-related artifacts are present in the image.

A total of 110 subjects (75 females) aged between 18 and 68 years (mean ± standard deviation = 28.06 ± 11.21 years) with no history of neurological or psychiatric diseases participated in the experiment. Data were acquired on a Siemens Magnetom Prisma 3 T MRI scanner (Siemens Healthcare, Erlangen, Germany) at the Brain Imaging Centre, Research Centre for Natural Sciences. All head elements of the standard Siemens 20-channel head-neck receiver coil were enabled during data acquisition. The protocol included T1-weighted 3D MPRAGE anatomical imaging using twofold in-plane GRAPPA acceleration (TR/TE/FA = 2300 ms/3.03 ms/9°; FOV = 256 mm; isotropic 1 mm spatial resolution).

For each subject, a T1-weighted MR image was collected under three different conditions, resulting in a total of 330 records. A measurement was taken under conventional circumstances (CONV), that is, subjects were instructed to lay still in the scanner while fixating on a fixation spot in the center of the screen on a grey background. In two other conditions, they were instructed to slightly nod their heads (tilt it down and then up once along the sagittal plane) once, whenever the instruction to do so appeared in the center of the screen. Either five (MOVE1) or ten (MOVE2) nods had to be performed in total. The interstimulus interval between the nodding instructions was constant in each of the conditions. Subjects briefly practiced nodding prior to the measurements. They were required to avoid lifting their heads from the scanner table while nodding and to try to return their heads to the original position after performing a nod as much as possible.

**Figure 3:**
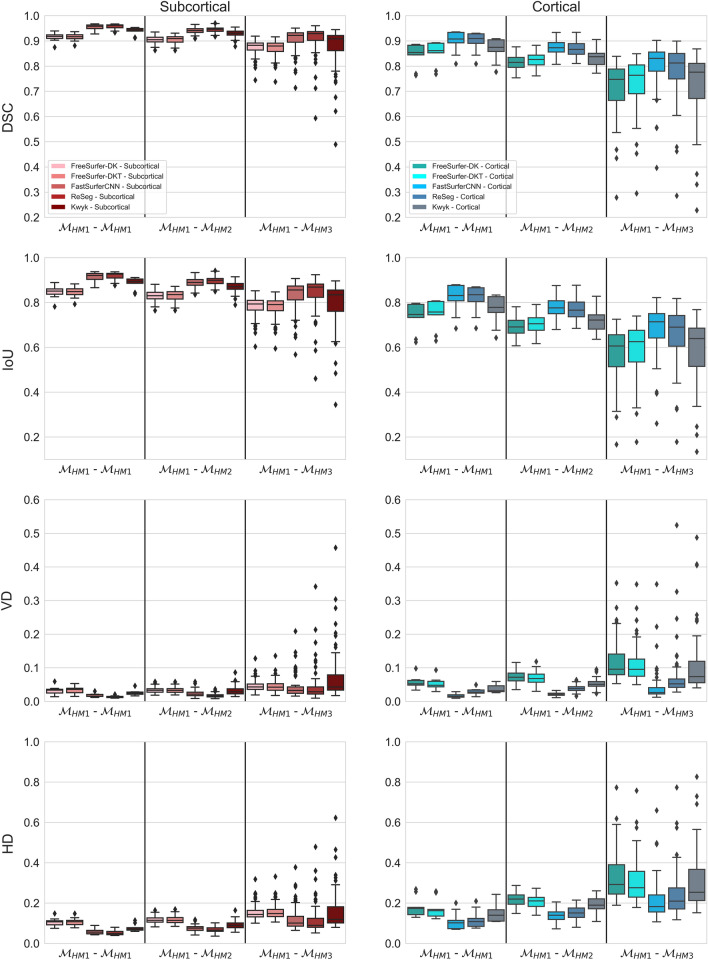
Boxplots showing the distributions of the dice similarity coefficient (DSC), intersection over union (IoU), volumetric difference (DF), and Hausdorff distance (HD) for FreeSurfer and deep learning-based segmentation methods. Evaluation metrics were calculated to compare the segmentation masks generated for good quality images acquired under rest to the masks generated for good (${M}_{HM1}$*—*${M}_{HM1}$), medium (${M}_{HM1}$*—*${M}_{HM2}$), and bad quality images (${M}_{HM1}$*—*${M}_{HM3}$) acquired under active head motion.

**Figure 4:**
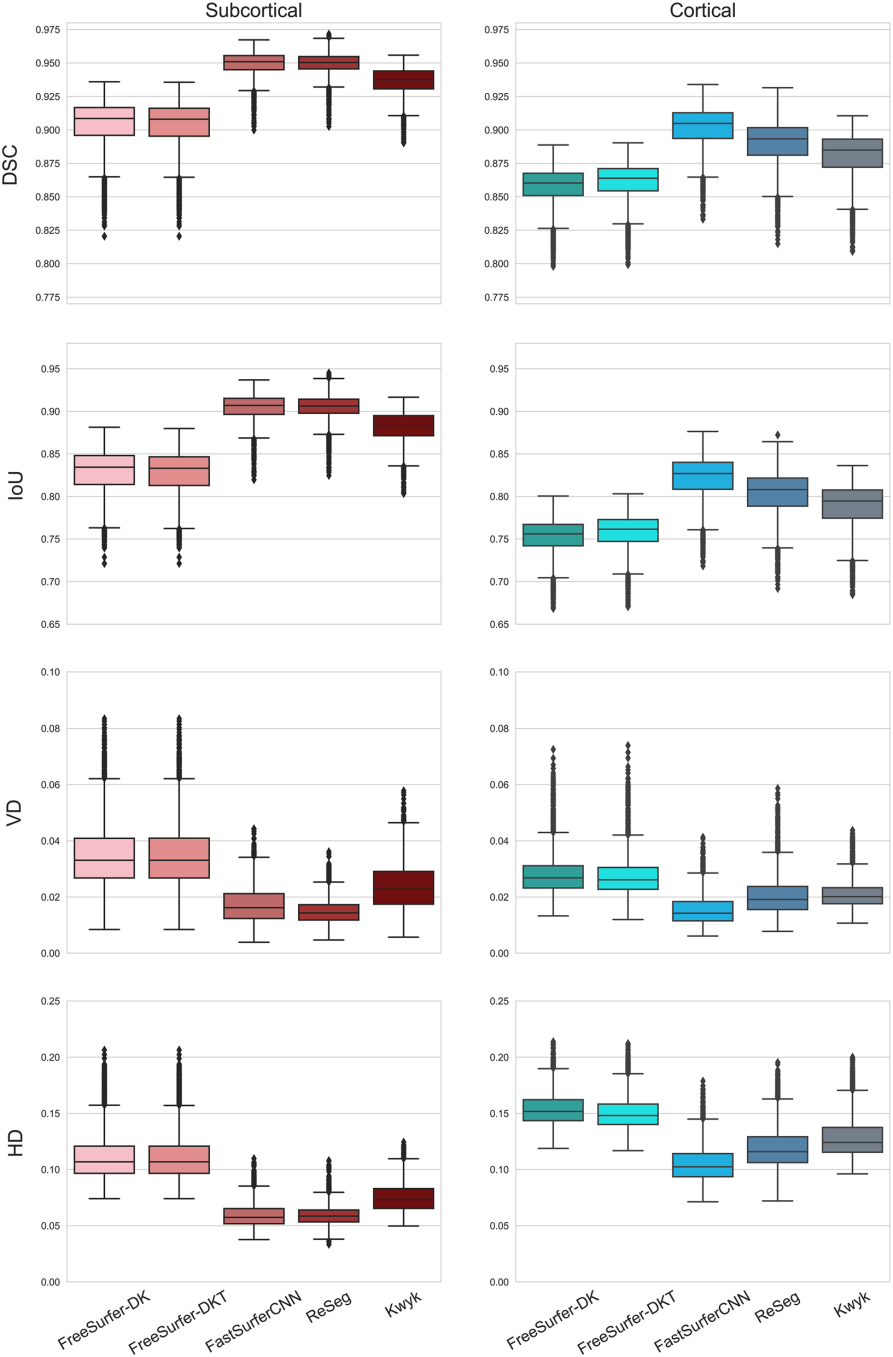
Boxplots showing the distributions of the dice similarity coefficient (DSC), intersection over union (IoU), volumetric difference (VD), and Hausdorff distance (HD) for FreeSurfer and deep learning-based segmentation methods. Evaluation metrics were calculated to compare segmentation masks generated for images from the same subjects in the test–retest dataset.

The extent of motion-related artifacts varied between subjects and conditions to a great extent. For this reason, each record was rated on a 4-point scale based on image quality. Rating was performed on the basis of visual inspection by five radiologists—two senior radiologists with more than ten years of experience and three junior radiologists with three years of experience. Senior radiologists trained junior radiologists and revised their ratings to ensure a consistent evaluation of image quality from the point of view of clinical diagnostic utility. By collapsing the ratings for the best and second-best quality images, the records were partitioned into three categories: clinically good (HM1), medium (HM2), and bad (HM3) quality images. For example images, see Figs. 3–5 in Appendix (the displayed MR images were deidentified by removing the facial features using the technique introduced by Bischoff-Grethe et al.^42^)*.* Six records were not rated due to a technical error and were excluded from the present analyses.

#### Test–retest dataset

We used the test–retest (TR) dataset^19^ to investigate the test–retest reliability of FreeSurfer and deep learning-based segmentation methods. This dataset contains 120 records acquired from 3 subjects in 20 sessions (2 records in each session) spanning 31 days. For each subject, we coregistered all the records to the first record using rigid-body transformation with the FSL FLIRT tool^43^ before segmentation. We used rigid-body transformation, assuming that structural changes in the brain are negligible within a period of 31 days.

#### Notations

In the following sections, we refer to the different sets of segmentation masks with superscript notations: FreeSurfer segmentation masks corresponding to the Desikan-Killiany atlas (${M}^{FS-DK}$) or the Desikan-Killiany-Tourville atlas (${M}^{FS-DKT}$), and the segmentation masks output by FastSurferCNN (${M}^{Fast}$), Kwyk (${M}^{Kwyk}$), or ReSeg (${M}^{ReSeg}$). We use subscript notations to refer to the sets of input MRI volumes from the Test–Retest (${M}_{TR}$) or the Head Motion dataset (${M}_{HM1}$–${M}_{HM3}$) for which the segmentation masks are generated. Thus, for example, the set of segmentation masks output by the ReSeg pipeline for the medium quality images from the Head Motion dataset is denoted by ${M}_{HM2}^{ReSeg}$.

### Data processing

Before feeding the raw MRI records into the deep learning models, they were resampled to 1 mm^3^ resolution with 3D 3rd order spline interpolation when necessary. The input of the segmentation pipeline is a 256 × 256 × 256 array, therefore the edges of the volumes were cropped or padded with zeros when the array was of different shape. Thenceforth the voxel intensities of the records were normalized to *N*(0, 1).

### Training and implementation

Net_Crop_ and Net_ReSeg_ were implemented in TensorFlow 2.2 and trained on 2 NVIDIA GeForce RTX 2080Ti GPUs with data parallelization. The two networks were trained separately with a mini-batch size of 8 volumes in the case of Net_Crop_ and with a mini-batch size of 2 volumes in the case of Net_ReSeg_. Both networks were trained using early stopping with the patience of 20 and 25 epochs for Net_Crop_ and Net_ReSeg_, respectively. Training Net_Crop_ using early stopping took 31 h while the training time of Net_ReSeg_ was nine and a half days. Inference time using the ReSeg pipeline was 11 s using a single GPU (NVIDIA GeForce RTX 2080Ti).

### Evaluation

#### Evaluation metrics

We used the Dice Similarity Coefficient (DSC), a commonly applied metric when evaluating medical image segmentations^31^, to quantify the overlap between binary ground truth and predicted segmentation masks. The Dice Similarity Coefficient ranges between 0 and 1, with higher scores indicating greater overlap between the segmentation maps. Besides the direct comparison of segmentation maps, DSC is frequently used to measure the reproducibility of segmentations^44^. We also used the Intersection over Union (IoU), also known as the Jaccard index^45^, to quantify the similarity between the ground truth and predicted segmentation maps. Similarly to the DSC, the IoU ranges between 0 and 1, with 1 indicating perfect overlap and 0 indicating no overlap at all between the segmentation maps.

Additionally, we employed the Hausdorff Distance (HD) metric which quantifies the spatial distance between two sets of points and is a recommended measure when the evaluation of segmentation boundaries is of particular importance^44,46^. In contrast to DSC and IoU, larger Hausdorff distance indicates less similarity between the ground truth and predicted segmentations. We also assessed the similarity between segmentations by calculating the absolute difference between their volumes according to the following formula:

3 $$VD\left({V}_{g}, {V}_{p}\right)=\frac{|{V}_{g} -{ V}_{p}|}{{V}_{g}}$$

where ${V}_{g}$ and ${V}_{p}$ denote the total volume of the voxels labelled as belonging to a particular brain region in the ground truth and predicted segmentations, respectively. When evaluating the generalization performance of the different deep learning methods (see “Generalizability”), FreeSurfer segmentations were used as ground truth. When assessing the sensitivity of the different methods to motion-induced artifacts (see “Evaluating sensitivity to motion artifacts using the head motion dataset”), the segmentations produced for the perfect quality images in the Head Motion dataset (D_HM1_) were used as ground truth. Finally, the segmentation produced for the image that had been recorded earlier was used as ground truth when comparing segmentations for image pairs from the same subjects to evaluate test–retest reliability (see “Evaluating test-retest reliability using the test–retest dataset”).

#### Generalizability

We examined the generalizability of the different deep learning methods by comparing their outputs to the segmentation masks generated by FreeSurfer for the good quality images from the Head Motion dataset (${M}_{HM1}$). Note that none of these records were used in the training of either the ReSeg pipeline or the other deep learning models; thus, they provide an independent dataset to assess the generalizability of the aforementioned methods. In order to exclusively compare segmentation masks that correspond to the same atlas, we compared ${M}_{HM1}^{ReSeg}$ and ${M}_{HM1}^{Kwyk}$ to ${M}_{HM1}^{FS-DK}$ and ${M}_{HM1}^{Fast}$ to ${M}_{HM1}^{FS-DKT}$ by calculating the evaluation metrics for the respective segmentations. Evaluation metrics were calculated for each brain structure separately, and then averaged separately for subjects, methods, and subcortical and cortical structures (referred to as ‘macro-regions’ in the following paragraphs).

#### Reliability

##### Evaluating sensitivity to motion artifacts using the head motion dataset

We used the Head Motion dataset to compare the reliability of FreeSurfer and deep learning-based segmentation methods across different levels of motion-induced artifacts. Subjects were included in the analysis if their CONV record received a score of 1 (good quality image). For each subject, the segmentation mask generated for the conventional HM1 record served as reference to which masks generated for MOVE1/MOVE2 images were compared. This way, we were able to form 11 pairs of ${M}_{HM1}$–${M}_{HM1}$, 75 pairs of ${M}_{HM1}-{M}_{HM2}$, and 91 pairs of ${M}_{HM1}-{M}_{HM3}$ segmentation masks for the three deep learning methods. FreeSurfer was unable to create masks for five HM3 records. Therefore, we were able to form 86 ${M}_{HM1}^{FS-DK}-{M}_{HM3}^{FS-DK}$ and ${M}_{HM1}^{FS-DKT}-{M}_{HM3}^{FS-DKT}$ mask pairs for evaluating FreeSurfer reliability (besides the 11 ${M}_{HM1}-{M}_{HM1}$ and 75 ${M}_{HM1}-{M}_{HM2}$ pairs that were used for evaluating the deep learning methods as well). Records belonging to the same subject were co-registered with FSL FLIRT^43^. Evaluation metrics were calculated for each pair of segmentation masks, separately for each brain region. They were then averaged across brain regions separately for each subject, method, macro-region, and type of pairing. Evaluation metrics for the deep learning-based methods were compared to FreeSurfer-DK/FreeSurfer-DKT using Wilcoxon signed-rank tests and Mann–Whitney U tests^47^. Comparisons were performed only within macro-regions and types of pairings.

##### Evaluating test–retest reliability using the test–retest dataset

Records in the Test–Retest dataset (TR) were used to assess the test–retest reliability of FreeSurfer and deep learning-based segmentation methods. The evaluation metrics were calculated for each brain region for every possible pair of segmentation masks within the same subject, separately for each segmentation method. Prior to statistical analysis, values of the evaluation metrics were averaged across record pairs, separately for each subject, macro-region, and segmentation method. Evaluation metrics for the deep learning-based methods were compared to FreeSurfer-DK/FreeSurfer-DKT using Wilcoxon signed-rank tests. Comparisons were performed only within macro-regions.

### Statistical analysis

All statistical tests were two-sided. *P*-values were corrected for multiple comparisons using the Benjamini–Hochberg procedure^48,49^. This correction procedure was performed for each evaluation metric separately. Differences were accepted as statistically significant if *p* < 0.05. All statistical tests were conducted in Python 3.6 using the Pingouin 0.3.10 statistical package^50^.

### Ethics statement

The research protocol used for collecting the Head Motion dataset was designed and conducted in accordance with the Hungarian regulations and laws, and was approved by the National Institute of Pharmacy and Nutrition (file number: OGYÉI/70184/2017). Data collection was carried out in the Brain Imaging Centre, Research Centre for Natural Sciences in Budapest, Hungary. The participants provided their written informed consent to participate in this study.

The study reported in this paper includes participants from the UK Biobank population cohort (https://www.ukbiobank.ac.uk/). The studies involving human participants were reviewed and approved by UK Biobank Research Ethics Committee (REC; approval number: 11/NW/0382).

## Results

### Generalizability

The distribution of the values of each evaluation metric for each deep learning-based segmentation method, with FreeSurfer masks used as the ground truth, are depicted in Fig. 2. On the whole, cortical segmentation appears to be a more challenging task than subcortical segmentation (median Dice score above 0.89 for all three deep learning methods in the latter case). Nevertheless, the median Dice score is above 0.8 for all three methods when segmenting cortical structures, showing the good generalization capability of deep learning-based brain segmentation methods when applied to MR images that are relatively free from motion-induced artifacts.

### Reliability

#### Sensitivity to motion artifacts

As expected, brain segmentation becomes less reliable with worsening MR image quality, as evidenced by the decrease in the similarity of brain segmentation masks when one of the input volumes becomes more and more corrupted by motion-related artifacts (Fig. 3). This drop in mask similarity is especially pronounced when comparing good quality images with bad ones (${M}_{HM1}$–${M}_{HM3}$). Figure 6 in Appendix shows examples of segmentation masks produced by the different methods. FastSurferCNN and ReSeg produced the most consistent segmentations of subcortical structures across different levels of motion-related artifacts. The median Dice similarity coefficient was 0.96 for both methods when comparing segmentation masks generated for good quality images (${M}_{HM1}-{M}_{HM1}$), 0.94 and 0.95 when comparing masks for good quality images to medium quality ones (${M}_{HM1}$–${M}_{HM2}$), and 0.92 and 0.93 when comparing masks for good quality images to those generated for bad quality ones (${M}_{HM1}$–${M}_{HM3}$) for FastSurferCNN and Reseg, respectively. In contrast, FreeSurfer achieved the lowest DSC values (0.92 for ${M}_{HM1}^{FS-DK}$–${M}_{HM1}^{FS-DK}$ and ${M}_{HM1}^{FS-DKT}$–${M}_{HM1}^{FS-DKT}$, 0.90 and 0.91 for ${M}_{HM1}^{FS-DK}-{M}_{HM2}^{FS-DK}$ and ${M}_{HM1}^{FS-DKT}-{M}_{HM2}^{FS-DKT}$, respectively, and 0.88 for ${M}_{HM1}^{FS-DK}-{M}_{HM3}^{FS-DK}$ and ${M}_{HM1}^{FS-DKT}-{M}_{HM3}^{FS-DKT}$), while Kwyk was in between (0.94 for ${M}_{HM1}^{Kwyk}-{M}_{HM1}^{Kwyk}$, 0.93 for ${M}_{HM1}^{Kwyk}-{M}_{HM2}^{Kwyk}$, and 0.91 for ${M}_{HM1}^{Kwyk}-{M}_{HM3}^{Kwyk}$) across all artifact levels. Importantly, all three deep learning-based methods produced significantly more similar segmentations (as reflected in DSC) than FreeSurfer, when comparing masks generated for good quality images to those produced for either good, medium, or bad quality ones (all *p* < 0.01). Note, however, that even for FreeSurfer, the median DSC was well above 0.8 for ${M}_{HM1}-{M}_{HM3}$, showing that reliable subcortical segmentation can be achieved for heavily artifact-corrupted images using this method as well. By and large, the pattern of results for IoU, HD and VD was highly similar to that observed in the case of DSC, with FastSurferCNN and ReSeg producing the most and FreeSurfer producing the least consistent segmentations with Kwyk in between, across all levels of motion-induced artifacts. One exception is the volumetric difference between good and bad quality image masks, in the case of which Kwyk performed on par with FreeSurfer (median VD = 0.043). Deep learning-based methods significantly outperformed FreeSurfer (all *p* < 0.05), except regarding VD, in the case of which there was no significant difference between Kwyk and FreeSurfer (all *p* > 0.13).

With regard to the segmentation of cortical structures, a highly similar pattern of results was observed as in the case of subcortical segmentation. FastSurferCNN and ReSeg achieved the best (highest DSC/IoU and lowest HD/VD) and FreeSurfer the worst median evaluation metric values, with Kwyk in between, across all three artifact levels. Deep learning-based methods significantly outperformed FreeSurfer (all *p* < 0.05), except for Kwyk, in the case of which there were no significant differences in DSC (*p* = 0.074) and IoU (*p* = 0.083) when comparing masks for good quality images to those generated for bad quality ones (${M}_{HM1}^{Kwyk}-{M}_{HM3}^{Kwyk}$).

#### Test–retest reliability

We examined the values of each evaluation metric for masks generated for repeated measurements of the same subjects using the Test–Retest dataset. According to our results, all deep learning-based methods had better test–retest reliability than FreeSurfer (Fig. 4). The differences between FreeSurfer and the other methods were significant for all similarity measures, in the case of cortical and subcortical segmentation as well (all *p* < 0.001).

## Discussion

In the present study, we investigated whether head motion-induced artifacts in MR images affect the consistency of whole-brain segmentation performed by FreeSurfer and deep learning-based segmentation methods to a similar extent. To this end, we collected brain scans from a large number of participants under rest and under two active head motion conditions, and divided these images into three different categories corresponding to different degrees of image quality (clinically good/medium/bad) based on the ratings of five radiologists. First, we established that the deep learning-based methods under scrutiny generalize well to the good quality images collected in our lab. This corroborates previous results showing that FastSurferCNN demonstrates comparable performance across different types of MR scanners and neurodegenerative disease states^18^, and that the Bayesian neural network referred to as Kwyk generalizes well to an out-of-site test set^16^. The results also provide evidence for the sound generalizability of our newly developed MRI processing pipeline called ReSeg, which consists of two convolutional neural networks performing the appropriate cropping of the input volume and subsequent whole-brain segmentation. Second, we assessed the consistency between the segmentations generated for the different quality images by comparing the masks generated for good quality images obtained under rest to those produced for either good, medium, or bad quality images obtained under active head motion. Compared to FreeSurfer, all three deep learning-based methods provided significantly more consistent segmentations across different levels of image quality. Thus, our results suggest that deep learning models can provide more reliable whole-brain segmentation than FreeSurfer even when image quality is severely diminished. A similar pattern of results was obtained using the brain scans from the Test–Retest dataset acquired under conventional circumstances, with deep learning models showing better consistency than FreeSurfer, in line with previous observations^18^.

Subject motion during magnetic resonance imaging is well known to introduce various artifacts to the image, and is known to reduce cortical gray matter volume and thickness estimations derived using traditional segmentation tools, including FreeSurfer^26,51,52^. Our results suggest that, compared to FreeSurfer, deep learning-based whole-brain segmentation methods may be less susceptible to motion-induced MR image artifacts. The deep learning models examined in the present work represent different computational approaches and implement different network architectures to whole-brain segmentation Thus, our results suggest that the observed effect is not specific to a particular type of neural network architecture. Nevertheless, there are still other successful approaches to whole-brain segmentation using deep learning that have not been investigated in the present study, such as employing a group of independent 3D U-Nets to process subvolumes^53,54^. Thus, the extent to which the relatively strong tolerance to motion-induced artifacts is a general property of deep learning-based brain segmentation methods is a matter of further investigation.

While convolutional neural networks are generally able to perform reasonably well when image quality is mildly degraded, evidence shows that blur and other distortions affecting image quality, such as Gaussian or salt-and-pepper noise, have a detrimental effect on the CNN-based classification^55–58^ and segmentation^59^ of images depicting everyday objects, especially when compared to human performance^60,61^. There is considerable variability in the extent to which different types of network architectures suffer from this problem^55,57,60^, showing that appropriate model selection may provide robustness against image artifacts. In fact, there are several options to improve the resiliency of deep learning models to the degradation of image quality. Invariance to image noise can be learned during training, for example by applying dropout in the input layer^62^. A state-of-the art network trained on high quality images can be fine-tuned on low quality ones. CNN-based semantic segmentation of blurred images has been shown to improve with fine-tuning, although a significant gap remained between the performance on sharp and blurred images^59^. In one study, CNNs trained directly on distorted images consistently outperformed human subjects in classification, although they generalized extremely poorly to images containing artifact types on which they had not been trained^61^. A promising solution to this problem is to use an ensemble of networks, with each network specializing in a specific type of distortion^63^. Thus, deep learning methods offer a range of options to deal with artifacts in image processing. While the studies reviewed above involved images displaying everyday objects, the methods discussed can be readily evaluated in the context of brain segmentation in the hope of further improving the reliability of deep learning models when processing artifact-corrupted images. These approaches are much more feasible than introducing algorithmic changes to traditional neuroimaging processing pipelines, which can be performed only by a handful of experts and often have unforeseen consequences^18^.

The present study involved a large number of subjects and applied quality control performed by five radiologists, which allowed for the comparison of the different segmentation methods from the point of view of clinical utility. Deep learning-based methods provided more consistent segmentation than FreeSurfer for medium quality images which are commonplace in clinical practice. Our results argue in favor of the practical applicability of deep learning-based methods for whole-brain segmentation, especially when studying brain structural alterations in neurological and neuropsychiatric disorders associated with an increased amount of movement.

## Supplementary Information

Supplementary Information.

## Data Availability

Data from ADNI^39^, OASIS3^41^ and SLIM^40^ is publicly available. Data from the UK Biobank^38^ is available by application. The Head Motion dataset will be shared with the wider research community in the near future as part of a separate publication that is currently being prepared. The code for running ReSeg will be available at https://gitlab.com/rcns-bic/reseg-whole-brain-segmentation upon publication.
