# Supplementary material for: Effect of head motion-induced artefacts on the reliability of deep learning-based whole-brain segmentation
Source: Sci Rep. 2022 Jan 31;12:1618. doi: 10.1038/s41598-022-05583-3 (PMC8803940; doi:10.1038/s41598-022-05583-3)
Supplement: Supplementary file 1 — Supplementary Information. [file 41598_2022_5583_MOESM1_ESM.docx]

| **Input name** | **Layer** | **Type** | **Output shape** |
| --- | --- | --- | --- |
|  | layer_0/input | INPUT | BATCHx76x76x92x1 |
| layer_0/input | layer_1/conv_1 | CONV(9x9x9@136) | BATCHx38x38x46x136 |
| layer_1/conv_1 | layer_1/conv_2 | CONV(7x7x7@272) | BATCHx38x38x46x272 |
| layer_1/conv_2 | layer_1/conv_3 | CONV(5x5x5@272) | BATCHx19x19x23x272 |
| layer_1/conv_3 | layer_1/batchnorm | BatchNorm | BATCHx19x19x23x272 |
| layer_1/batchnorm | layer_1/activation | Activation (Swish) | BATCHx19x19x23x272 |
| layer_1/activation | layer_2/conv_1 | CONV(1x1x1@136) | BATCHx19x19x23x136 |
| layer_2/conv_1 | layer_2/conv_2 | CONV(3x3x3@272) | BATCHx19x19x23x272 |
| layer_2/conv_2 | layer_2/conv_3 | CONV(1x1x1@272) | BATCHx19x19x23x272 |
| layer_2/conv_3 | layer_2/conv_4 | CONV(3x3x3@544) | BATCHx10x10x12x544 |
| layer_2/conv_4 | layer_2/batchnorm | BatchNorm | BATCHx10x10x12x544 |
| layer_2/batchnorm | layer_2/activation | Activation (Swish) | BATCHx10x10x12x544 |
| layer_2/activation | layer_3/conv_1 | CONV(1x1x1@272) | BATCHx10x10x12x272 |
| layer_3/conv_1 | layer_3/conv_2 | CONV(3x3x3@544) | BATCHx10x10x12x544 |
| layer_3/conv_2 | layer_3/conv_3 | CONV(1x1x1@272) | BATCHx10x10x12x272 |
| layer_3/conv_3 | layer_3/conv_4 | CONV(3x3x3@544) | BATCHx10x10x12x544 |
| layer_3/conv_4 | layer_3/batchnorm_1 | BatchNorm | BATCHx10x10x12x544 |
| layer_3/batchnorm_1 | layer_3/activation_1 | Activation (Swish) | BATCHx10x10x12x544 |
| layer_3/activation_1 | layer_3/conv_5 | CONV(1x1x1@272) | BATCHx10x10x12x272 |
| layer_3/conv_5 | layer_3/conv_6 | CONV(3x3x3@544) | BATCHx10x10x12x544 |
| layer_3/conv_6 | layer_3/conv_7 | CONV(1x1x1@544) | BATCHx10x10x12x544 |
| layer_3/conv_7 | layer_3/conv_8 | CONV(3x3x3@1088) | BATCHx10x10x12x1088 |
| layer_3/conv_8 | layer_3/batchnorm_2 | BatchNorm | BATCHx10x10x12x1088 |
| layer_3/batchnorm_2 | layer_3/activation_2 | Activation (Swish) | BATCHx10x10x12x1088 |
| layer_3/activation_2 | layer_4/conv_1 | CONV(3x3x3@544) | BATCHx10x10x12x544 |
| layer_2/activation; layer_4/conv_1 | layer_4/concat_1 | Concatenation | BATCHx10x10x12x1088 |
| layer_4/concat_1 | layer_4/conv_2 | DECONV(2x2x2@272) | BATCHx19x19x23x272 |
| layer_1/activation; layer_4/conv_2 | layer_4/concat_2 | Concatenation | BATCHx19x19x23x544 |
| layer_4/concat_2 | layer_5/conv_1 | CONV(1x1x1@272) | BATCHx19x19x23x272 |
| layer_5/conv_1 | layer_5/conv_2 | CONV(3x3x3@136) | BATCHx19x19x23x136 |
| layer_5/conv_2 | layer_5/conv_3 | CONV(1x1x1@272) | BATCHx19x19x23x272 |
| layer_5/conv_3 | layer_5/conv_4 | DECONV(2x2x2@272) | BATCHx38x38x46x272 |
| layer_5/conv_4 | layer_5/batchnorm | BatchNorm | BATCHx38x38x46x272 |
| layer_5/batchnorm | layer_5/activation | Activation (Swish) | BATCHx38x38x46x272 |
| layer_1/conv_1; layer_5/activation | layer_5/concat | Concatenation | BATCHx38x38x46x408 |
| layer_5/concat | layer_6/conv_1 | CONV(1x1x1@136) | BATCHx38x38x46x136 |
| layer_6/conv_1 | layer_6/conv_2 | CONV(3x3x3@68) | BATCHx38x38x46x68 |
| layer_6/conv_2 | layer_6/conv_3 | CONV(1x1x1@136) | BATCHx38x38x46x136 |
| layer_6/conv_3 | layer_6/conv_4 | DECONV(2x2x2@136) | BATCHx76x76x92x136 |
| layer_6/conv_4 | layer_6/batchnorm | BatchNorm | BATCHx76x76x92x136 |
| layer_6/batchnorm | layer_6/activation | Activation (Swish) | BATCHx76x76x92x136 |
| layer_6/activation | layer_7/conv_1 | CONV(1x1x1@136) | BATCHx76x76x92x136 |
| layer_7/conv_1; layer_0/input | layer_7/concat | Concatenation | BATCHx76x76x92x137 |
| layer_7/concat | output | CONV(1x1x1@50) | BATCHx76x76x92x50 |

Table 1. Architecture of neural network Net_ReSeg_ that performs whole-brain segmentation on the cropped MRI volume.

| FreeSurfer-DK label | New label | Brain region |  | FreeSurfer-DK label | New label | Brain region |
| --- | --- | --- | --- | --- | --- | --- |
| 0 | 0 | Unknown |  | 1001 | 16 | ctx-lh-bankssts |
| 2 | 1 | Left-Cerebral-White-Matter |  | 1002 | 17 | ctx-lh-caudalanteriorcingulate |
| 4 | 2 | Left-Lateral-Ventricle |  | 1003 | 18 | ctx-lh-caudalmiddlefrontal |
| 5 | 2 | Left-Inf-Lat-Vent |  | 1005 | 19 | ctx-lh-cuneus |
| 7 | 3 | Left-Cerebellum-White-Matter |  | 1006 | 20 | ctx-lh-entorhinal |
| 8 | 4 | Left-Cerebellum-Cortex |  | 1007 | 21 | ctx-lh-fusiform |
| 10 | 5 | Left-Thalamus-Proper |  | 1008 | 22 | ctx-lh-inferiorparietal |
| 11 | 6 | Left-Caudate |  | 1009 | 23 | ctx-lh-inferiortemporal |
| 12 | 7 | Left-Putamen |  | 1010 | 24 | ctx-lh-isthmuscingulate |
| 13 | 8 | Left-Pallidum |  | 1011 | 25 | ctx-lh-lateraloccipital |
| 14 | 2 | 3rd-Ventricle |  | 1012 | 26 | ctx-lh-lateralorbitofrontal |
| 15 | 2 | 4th-Ventricle |  | 1013 | 27 | ctx-lh-lingual |
| 16 | 9 | Brain-Stem |  | 1014 | 28 | ctx-lh-medialorbitofrontal |
| 17 | 10 | Left-Hippocampus |  | 1015 | 29 | ctx-lh-middletemporal |
| 18 | 11 | Left-Amygdala |  | 1016 | 30 | ctx-lh-parahippocampal |
| 24 | 12 | CSF |  | 1017 | 31 | ctx-lh-paracentral |
| 26 | 13 | Left-Accumbens-area |  | 1018 | 32 | ctx-lh-parsopercularis |
| 28 | 14 | Left-VentralDC |  | 1019 | 33 | ctx-lh-parsorbitalis |
| 41 | 1 | Right-Cerebral-White-Matter |  | 1020 | 34 | ctx-lh-parstriangularis |
| 43 | 2 | Right-Lateral-Ventricle |  | 1021 | 35 | ctx-lh-pericalcarine |
| 44 | 2 | Right-Inf-Lat-Vent |  | 1022 | 36 | ctx-lh-postcentral |
| 46 | 3 | Right-Cerebellum-White-Matter |  | 1023 | 37 | ctx-lh-posteriorcingulate |
| 47 | 4 | Right-Cerebellum-Cortex |  | 1024 | 38 | ctx-lh-precentral |
| 49 | 5 | Right-Thalamus-Proper |  | 1025 | 39 | ctx-lh-precuneus |
| 50 | 6 | Right-Caudate |  | 1026 | 40 | ctx-lh-rostralanteriorcingulate |
| 51 | 7 | Right-Putamen |  | 1027 | 41 | ctx-lh-rostralmiddlefrontal |
| 52 | 8 | Right-Pallidum |  | 1028 | 42 | ctx-lh-superiorfrontal |
| 53 | 10 | Right-Hippocampus |  | 1029 | 43 | ctx-lh-superiorparietal |
| 54 | 11 | Right-Amygdala |  | 1030 | 44 | ctx-lh-superiortemporal |
| 58 | 13 | Right-Accumbens-area |  | 1031 | 45 | ctx-lh-supramarginal |
| 60 | 14 | Right-VentralDC |  | 1032 | 46 | ctx-lh-frontalpole |
| 72 | 2 | 5th-Ventricle |  | 1033 | 47 | ctx-lh-temporalpole |
| 192 | 15 | Corpus_Callosum |  | 1034 | 48 | ctx-lh-transversetemporal |
| 251 | 15 | CC_Posterior |  | 1035 | 49 | ctx-lh-insula |
| 252 | 15 | CC_Mid_Posterior |  | 2001 | 16 | ctx-rh-bankssts |
| 253 | 15 | CC_Central |  | 2002 | 17 | ctx-rh-caudalanteriorcingulate |
| 254 | 15 | CC_Mid_Anterior |  | 2003 | 18 | ctx-rh-caudalmiddlefrontal |
| 255 | 15 | CC_Anterior |  | 2005 | 19 | ctx-rh-cuneus |
|  |  |  |  | 2006 | 20 | ctx-rh-entorhinal |
|  |  |  |  | 2007 | 21 | ctx-rh-fusiform |
|  |  |  |  | 2008 | 22 | ctx-rh-inferiorparietal |
|  |  |  |  | 2009 | 23 | ctx-rh-inferiortemporal |
|  |  |  |  | 2010 | 24 | ctx-rh-isthmuscingulate |
|  |  |  |  | 2011 | 25 | ctx-rh-lateraloccipital |
|  |  |  |  | 2012 | 26 | ctx-rh-lateralorbitofrontal |
|  |  |  |  | 2013 | 27 | ctx-rh-lingual |
|  |  |  |  | 2014 | 28 | ctx-rh-medialorbitofrontal |
|  |  |  |  | 2015 | 29 | ctx-rh-middletemporal |
|  |  |  |  | 2016 | 30 | ctx-rh-parahippocampal |
|  |  |  |  | 2017 | 31 | ctx-rh-paracentral |
|  |  |  |  | 2018 | 32 | ctx-rh-parsopercularis |
|  |  |  |  | 2019 | 33 | ctx-rh-parsorbitalis |
|  |  |  |  | 2020 | 34 | ctx-rh-parstriangularis |
|  |  |  |  | 2021 | 35 | ctx-rh-pericalcarine |
|  |  |  |  | 2022 | 36 | ctx-rh-postcentral |
|  |  |  |  | 2023 | 37 | ctx-rh-posteriorcingulate |
|  |  |  |  | 2024 | 38 | ctx-rh-precentral |
|  |  |  |  | 2025 | 39 | ctx-rh-precuneus |
|  |  |  |  | 2026 | 40 | ctx-rh-rostralanteriorcingulate |
|  |  |  |  | 2027 | 41 | ctx-rh-rostralmiddlefrontal |
|  |  |  |  | 2028 | 42 | ctx-rh-superiorfrontal |
|  |  |  |  | 2029 | 43 | ctx-rh-superiorparietal |
|  |  |  |  | 2030 | 44 | ctx-rh-superiortemporal |
|  |  |  |  | 2031 | 45 | ctx-rh-supramarginal |
|  |  |  |  | 2032 | 46 | ctx-rh-frontalpole |
|  |  |  |  | 2033 | 47 | ctx-rh-temporalpole |
|  |  |  |  | 2034 | 48 | ctx-rh-transversetemporal |
|  |  |  |  | 2035 | 49 | ctx-rh-insula |

Table 2. Mapping between FreeSurfer labels in the Desikan-Killiany (DK) atlas and the labels used in the present study. These ‘new labels’ constitute the segmentation labels applied by Kwyk and ReSeg, and they were also used to evaluate the reliability of FreeSurfer segmentations according to the DK atlas. Cortical regions are denoted by the ‘ctx-’ prefix. Left and right hemisphere cortical structures are denoted by the ‘lh-’ and ‘rh-’ prefixes, respectively.

| FreeSurfer-DKT label | New label | Brain region |  | FreeSurfer-DKT label | New label | Brain region |
| --- | --- | --- | --- | --- | --- | --- |
| 0 | 0 | Unknown |  | 1002 | 17 | ctx-lh-caudalanteriorcingulate |
| 2 | 1 | Left-Cerebral-White-Matter |  | 1003 | 18 | ctx-lh-caudalmiddlefrontal |
| 4 | 2 | Left-Lateral-Ventricle |  | 1005 | 19 | ctx-lh-cuneus |
| 5 | 2 | Left-Inf-Lat-Vent |  | 1006 | 20 | ctx-lh-entorhinal |
| 7 | 3 | Left-Cerebellum-White-Matter |  | 1007 | 21 | ctx-lh-fusiform |
| 8 | 4 | Left-Cerebellum-Cortex |  | 1008 | 22 | ctx-lh-inferiorparietal |
| 10 | 5 | Left-Thalamus-Proper |  | 1009 | 23 | ctx-lh-inferiortemporal |
| 11 | 6 | Left-Caudate |  | 1010 | 24 | ctx-lh-isthmuscingulate |
| 12 | 7 | Left-Putamen |  | 1011 | 25 | ctx-lh-lateraloccipital |
| 13 | 8 | Left-Pallidum |  | 1012 | 26 | ctx-lh-lateralorbitofrontal |
| 14 | 2 | 3rd-Ventricle |  | 1013 | 27 | ctx-lh-lingual |
| 15 | 2 | 4th-Ventricle |  | 1014 | 28 | ctx-lh-medialorbitofrontal |
| 16 | 9 | Brain-Stem |  | 1015 | 29 | ctx-lh-middletemporal |
| 17 | 10 | Left-Hippocampus |  | 1016 | 30 | ctx-lh-parahippocampal |
| 18 | 11 | Left-Amygdala |  | 1017 | 31 | ctx-lh-paracentral |
| 24 | 12 | CSF |  | 1018 | 32 | ctx-lh-parsopercularis |
| 26 | 13 | Left-Accumbens-area |  | 1019 | 33 | ctx-lh-parsorbitalis |
| 28 | 14 | Left-VentralDC |  | 1020 | 34 | ctx-lh-parstriangularis |
| 41 | 1 | Right-Cerebral-White-Matter |  | 1021 | 35 | ctx-lh-pericalcarine |
| 43 | 2 | Right-Lateral-Ventricle |  | 1022 | 36 | ctx-lh-postcentral |
| 44 | 2 | Right-Inf-Lat-Vent |  | 1023 | 37 | ctx-lh-posteriorcingulate |
| 46 | 3 | Right-Cerebellum-White-Matter |  | 1024 | 38 | ctx-lh-precentral |
| 47 | 4 | Right-Cerebellum-Cortex |  | 1025 | 39 | ctx-lh-precuneus |
| 49 | 5 | Right-Thalamus-Proper |  | 1026 | 40 | ctx-lh-rostralanteriorcingulate |
| 50 | 6 | Right-Caudate |  | 1027 | 41 | ctx-lh-rostralmiddlefrontal |
| 51 | 7 | Right-Putamen |  | 1028 | 42 | ctx-lh-superiorfrontal |
| 52 | 8 | Right-Pallidum |  | 1029 | 43 | ctx-lh-superiorparietal |
| 53 | 10 | Right-Hippocampus |  | 1030 | 44 | ctx-lh-superiortemporal |
| 54 | 11 | Right-Amygdala |  | 1031 | 45 | ctx-lh-supramarginal |
| 58 | 13 | Right-Accumbens-area |  | 1034 | 48 | ctx-lh-transversetemporal |
| 60 | 14 | Right-VentralDC |  | 1035 | 49 | ctx-lh-insula |
| 72 | 2 | 5th-Ventricle |  | 2002 | 17 | ctx-rh-caudalanteriorcingulate |
|  |  |  |  | 2003 | 18 | ctx-rh-caudalmiddlefrontal |
|  |  |  |  | 2005 | 19 | ctx-rh-cuneus |
|  |  |  |  | 2006 | 20 | ctx-rh-entorhinal |
|  |  |  |  | 2007 | 21 | ctx-rh-fusiform |
|  |  |  |  | 2008 | 22 | ctx-rh-inferiorparietal |
|  |  |  |  | 2009 | 23 | ctx-rh-inferiortemporal |
|  |  |  |  | 2010 | 24 | ctx-rh-isthmuscingulate |
|  |  |  |  | 2011 | 25 | ctx-rh-lateraloccipital |
|  |  |  |  | 2012 | 26 | ctx-rh-lateralorbitofrontal |
|  |  |  |  | 2013 | 27 | ctx-rh-lingual |
|  |  |  |  | 2014 | 28 | ctx-rh-medialorbitofrontal |
|  |  |  |  | 2015 | 29 | ctx-rh-middletemporal |
|  |  |  |  | 2016 | 30 | ctx-rh-parahippocampal |
|  |  |  |  | 2017 | 31 | ctx-rh-paracentral |
|  |  |  |  | 2018 | 32 | ctx-rh-parsopercularis |
|  |  |  |  | 2019 | 33 | ctx-rh-parsorbitalis |
|  |  |  |  | 2020 | 34 | ctx-rh-parstriangularis |
|  |  |  |  | 2021 | 35 | ctx-rh-pericalcarine |
|  |  |  |  | 2022 | 36 | ctx-rh-postcentral |
|  |  |  |  | 2023 | 37 | ctx-rh-posteriorcingulate |
|  |  |  |  | 2024 | 38 | ctx-rh-precentral |
|  |  |  |  | 2025 | 39 | ctx-rh-precuneus |
|  |  |  |  | 2026 | 40 | ctx-rh-rostralanteriorcingulate |
|  |  |  |  | 2027 | 41 | ctx-rh-rostralmiddlefrontal |
|  |  |  |  | 2028 | 42 | ctx-rh-superiorfrontal |
|  |  |  |  | 2029 | 43 | ctx-rh-superiorparietal |
|  |  |  |  | 2030 | 44 | ctx-rh-superiortemporal |
|  |  |  |  | 2031 | 45 | ctx-rh-supramarginal |
|  |  |  |  | 2034 | 48 | ctx-rh-transversetemporal |
|  |  |  |  | 2035 | 49 | ctx-rh-insula |

Table 3. Mapping between FreeSurfer labels in the Desikan-Killiany-Tourville (DKT) atlas and the labels used in the present study. These ‘new labels’ were used to evaluate the generalizability and reliability of FastSurferCNN, as well as the reliability of the segmentations produced by FreeSurfer according to the DKT atlas. Cortical regions are denoted by the ‘ctx-’ prefix. Left and right hemisphere cortical structures are denoted by the ‘lh-’ and ‘rh-’ prefixes, respectively.

| Training dataset | Number of records | Number of subjects | Mean age ± standard deviation (years) | Number of male subjects /records | Number of female subjects /records |
| --- | --- | --- | --- | --- | --- |
| UK Biobank | 560 | 560 | 60.27 ± 6.57 | 281/281 | 279/279 |
| OASIS3 | 41 | 38 | 71.61 ± 7.45 | 12/13 | 26/28 |
| SLIM | 411 | 326 | 20.69 ± 1.27 | 147/192 | 179/219 |
| ADNI | 460 | 372 | 74.08 ± 8.59 | 168/210 | 204/250 |

Table 4. Characteristics of the dataset used for the training of the ReSeg brain segmentation pipeline.

| Validation dataset | Number of records | Number of subjects | Mean age ± standard deviation (years) | Number of male subjects /records | Number of female subjects /records |
| --- | --- | --- | --- | --- | --- |
| UK Biobank | 107 | 107 | 59.15 ± 7.58 | 46/46 | 61/61 |
| OASIS3 | 11 | 11 | 73.27 ± 7.81 | 4/4 | 7/7 |
| SLIM | 112 | 106 | 20.92 ± 1.69 | 49/50 | 57/62 |
| ADNI | 85 | 83 | 75.02 ± 6.96 | 38/38 | 45/47 |

Table 5. Characteristics of the dataset used for the validation of the ReSeg brain segmentation pipeline.

| Evaluation dataset | Number of records | Number of subjects | Mean age ± standard deviation (years) | Number of male subjects /records | Number of female subjects /records |
| --- | --- | --- | --- | --- | --- |
| UK Biobank | 113 | 113 | 59.95 ± 6.61 | 48/48 | 65/65 |
| OASIS3 | 9 | 9 | 76.11 ± 6.88 | 7/7 | 2/2 |
| SLIM | 97 | 95 | 20.46 ± 1.55 | 40/40 | 55/57 |
| ADNI | 97 | 94 | 75.28 ± 7.21 | 44/47 | 50/50 |

Table 6. Characteristics of the dataset used for the evaluation of the ReSeg brain segmentation pipeline.


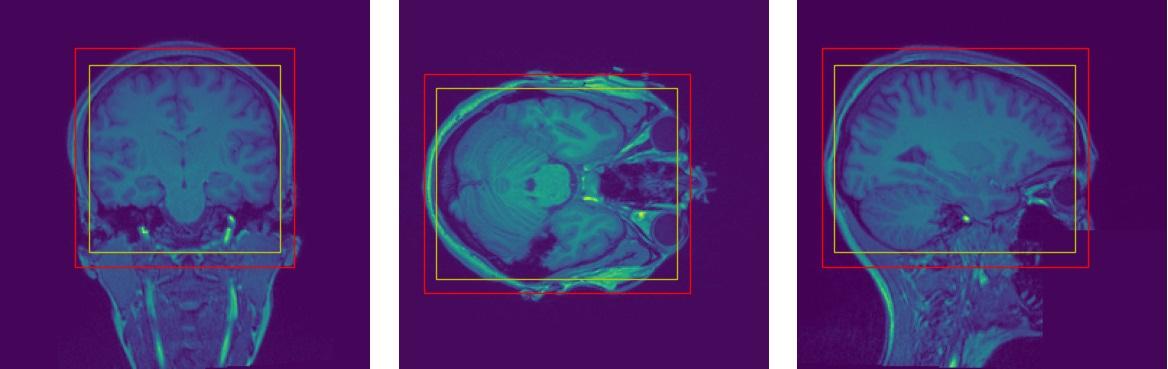


Figure 1. Coronal (left panel), axial (middle panel), and sagittal (right panel) slices from a clinically good quality MRI scan (HM1) in the Head Motion dataset. Yellow rectangles represent the bounding box predicted by Net_Crop_. Red rectangles represent the final bounding box, which was of the same size for all MRI scans (152 × 152 × 184 voxels) and was defined using the center point predicted by Net_Crop_ and the typical dimensions of adult human brains.


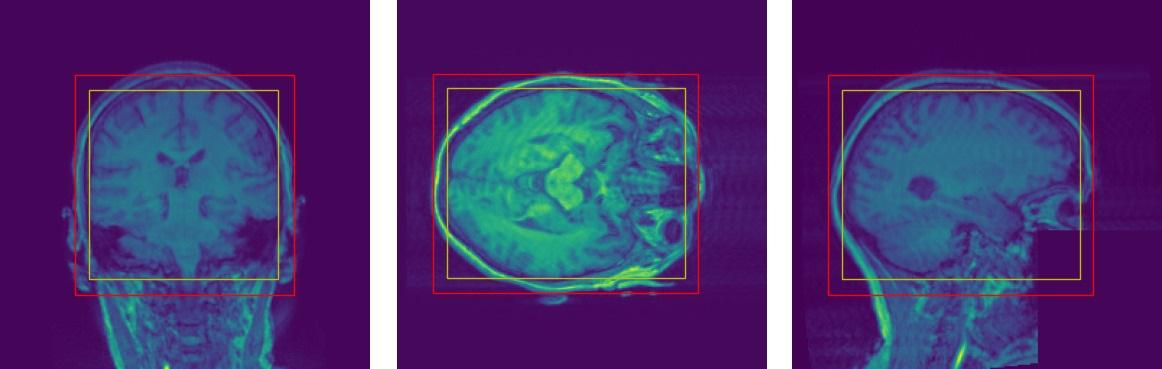


Figure 2. Coronal (left panel), axial (middle panel), and sagittal (right panel) slices from a clinically bad quality MRI scan (HM3) in the Head Motion dataset. Yellow rectangles represent the bounding box predicted by Net_Crop_. Red rectangles represent the final bounding box, which was of the same size for all MRI scans (152 × 152 × 184 voxels) and was defined using the center point predicted by Net_Crop_ and the typical dimensions of adult human brains.


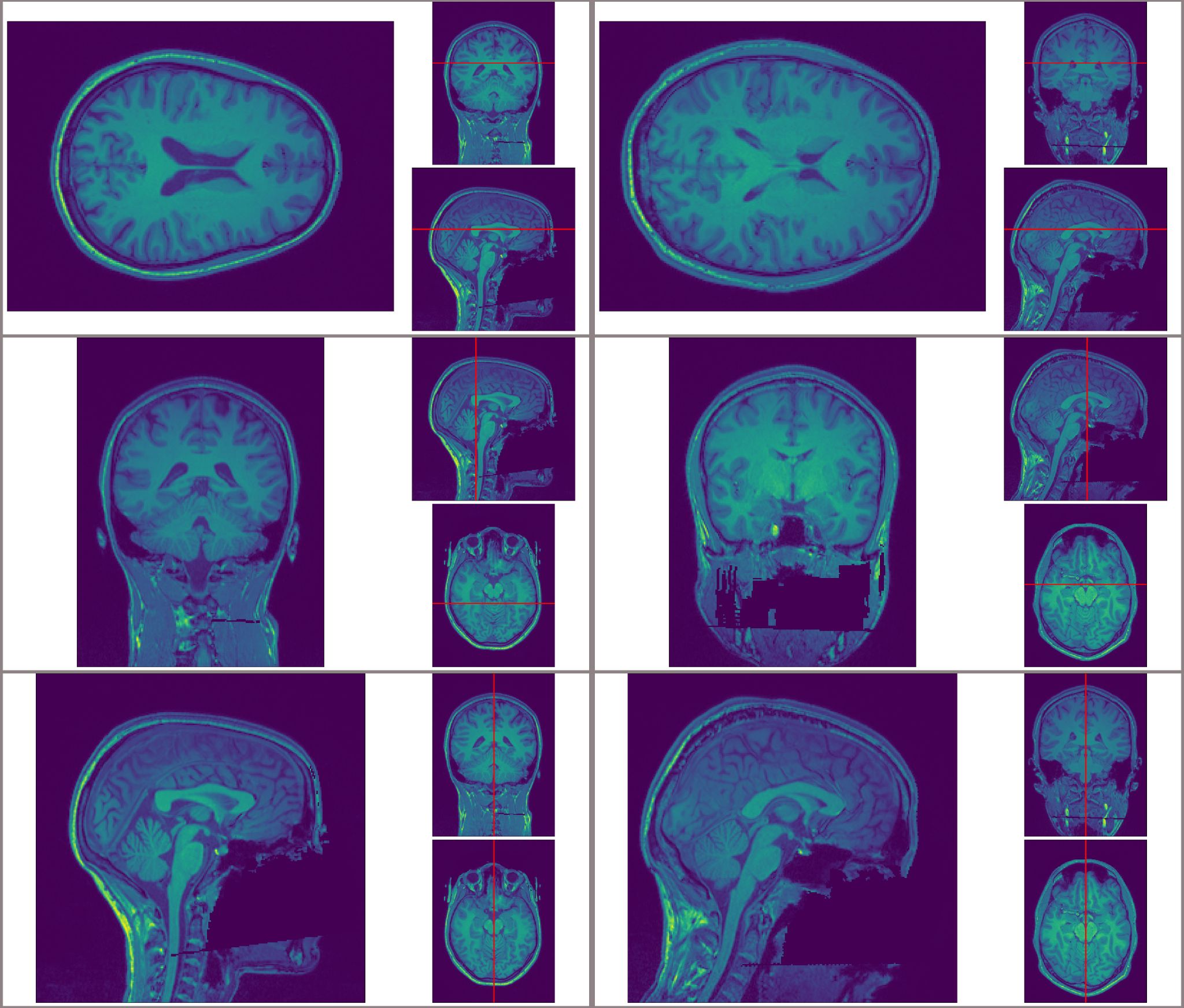


Figure 3. Clinically good quality (HM1) MR images from the Head Motion dataset from two subjects (the first subject on the left, the second subject on the right). Red lines in the insets highlight the location of each of the axial (upper row), coronal (middle row), and sagittal (lower row) slices.


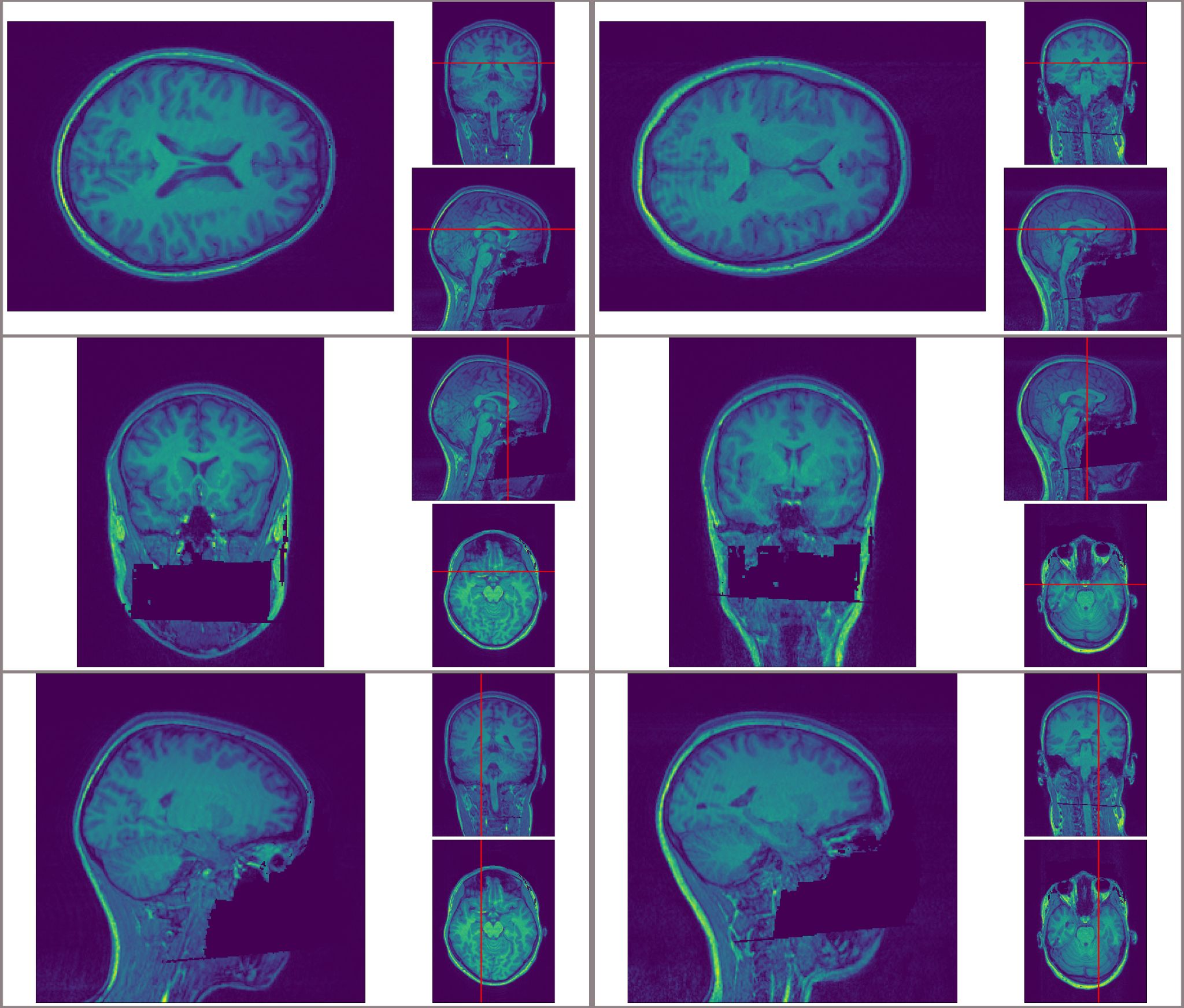


Figure 4. Clinically medium quality (HM2) MR images from the Head Motion dataset from two subjects (the first subject on the left, the second subject on the right). Red lines in the insets highlight the location of each of the axial (upper row), coronal (middle row), and sagittal (lower row) slices.


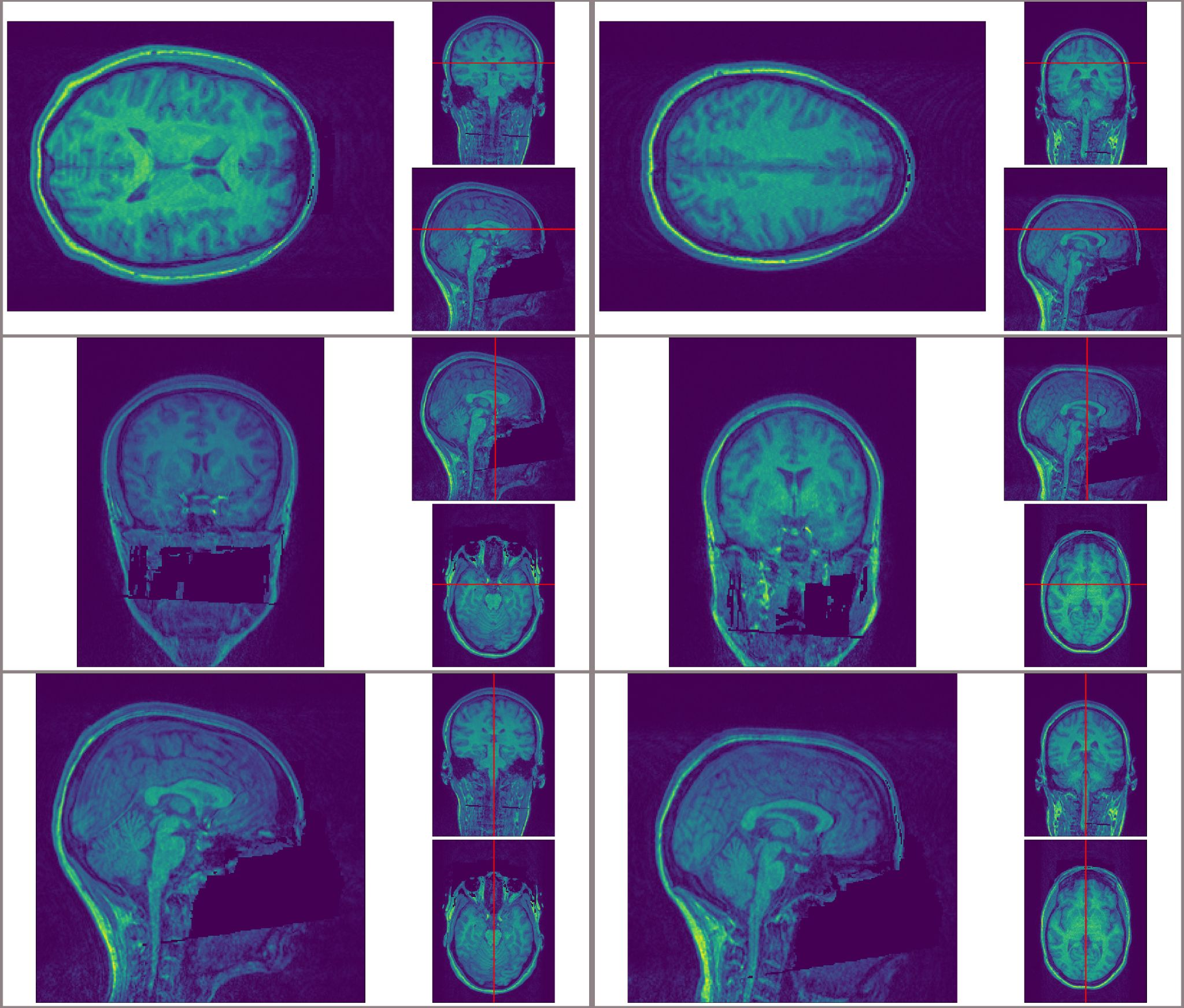


Figure 5. Clinically bad quality (HM3) MR images from the Head Motion dataset from two subjects (the first subject on the left, the second subject on the right). Red lines in the insets highlight the location of each of the axial (upper row), coronal (middle row), and sagittal (lower row) slices.


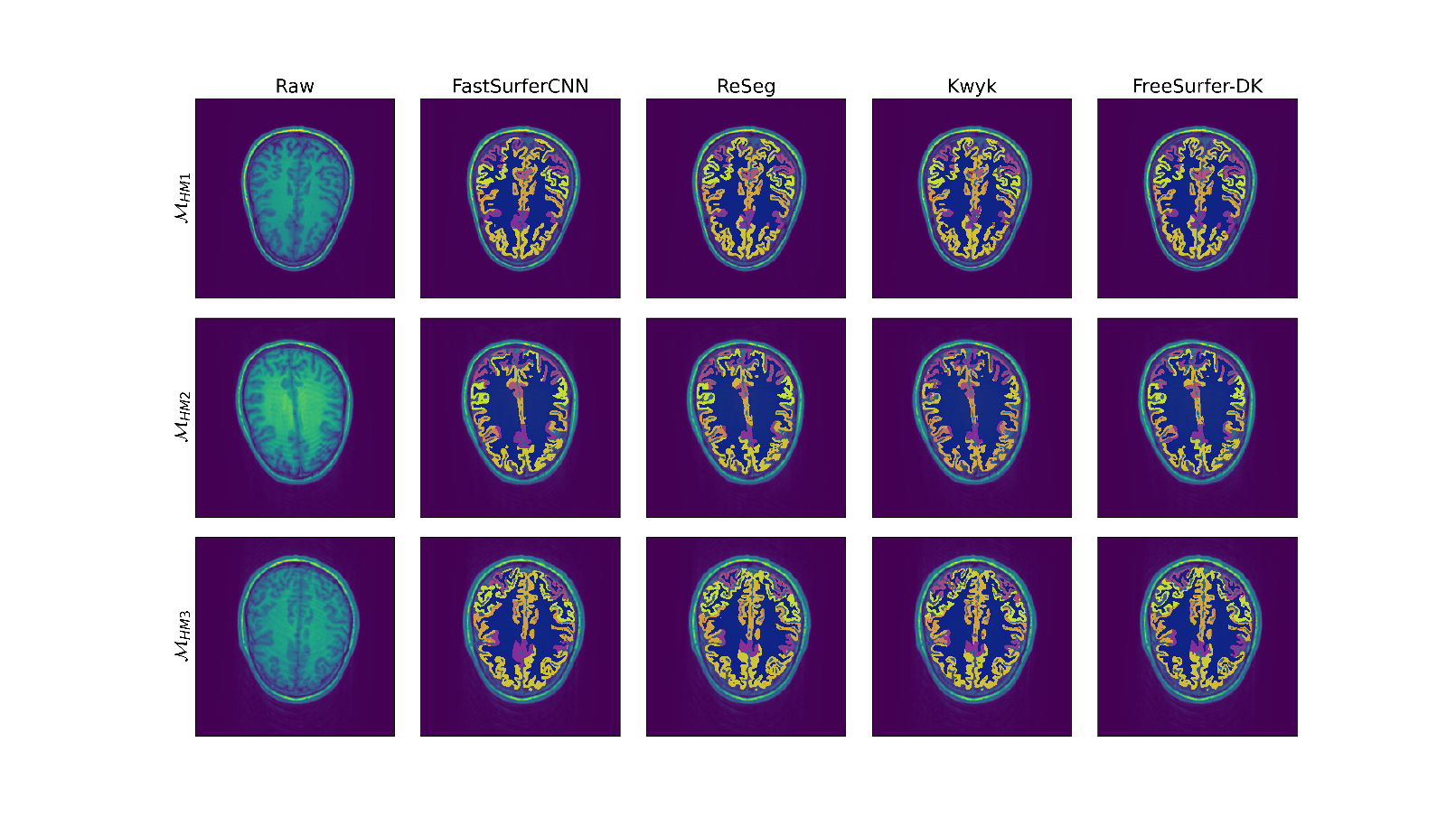
Figure 6. Axial slices from a clinically good (upper row), medium quality (middle row) and clinically bad quality (lower row) MRI scan in the Head Motion dataset. The first column shows the raw slices, the remaining columns show the segmentation masks produced by the corresponding methods overlaid on the raw slices.
